# Phylogenomics Clarifies Plastome Reduction and Phylogenetic Relationships in Monotropoideae

**DOI:** 10.1002/ece3.74449

**Published:** 2026-10-02

**Authors:** Guocui Deng, Kaili Duan, Niyan Xiang, Feng Gao, Peng Zhan, Rui Shu, Qingsong Lin, Qing Zuo, Tao Yuan

**Affiliations:** ^1^ College of Life Science and Technology Xinjiang University Ürümqi China; ^2^ Xinjiang Key Laboratory of Biological Resources and Genetic Engineering Ürümqi China; ^3^ State Key Laboratory of Hybrid Rice, Laboratory of Plant Systematics and Evolutionary Biology, College of Life Sciences Wuhan University Wuhan China; ^4^ School of Resources and Environmental Science Hubei University Wuhan China; ^5^ Hubei Provincial Key Laboratory for Protection and Application of Special Plant Germplasm in Wuling Area of China, College of Life Sciences South‐Central Minzu University Wuhan China; ^6^ Key Lab for Biology of Crop Pathogens and Insect Pests and Their Ecological Regulation of Zhejiang Province, College of Advanced Agricultural Sciences Zhejiang Agriculture & Forestry University Hangzhou Zhejiang China; ^7^ Institute of Intelligent Chinese Medicine Chongqing University of Chinese Medicine Chongqing College of Traditional Chinese Medicine Chongqing China

**Keywords:** evolution, *Monotropa callistoma*, Monotropoideae, phylogenetic analysis, plastome

## Abstract

Plastome evolution and biogeographic history remain poorly understood in mycoheterotrophic lineages. Here, we sequenced and assembled the complete plastome of *Monotropa callistoma* (Ericaceae), a fully mycoheterotrophic species, and compared it with other mycoheterotrophic plastomes. The *M. callistoma* plastome is 29,784 bp long, lacks the canonical quadripartite structure, and contains 40 genes, representing an intermediate level of reduction within Monotropoideae. Comparative analyses revealed extreme contraction in some lineages, including *Sciaphila* (12.8–21.5 kb), and a weak positive correlation between GC content and genome size. Within Monotropoideae, plastomes become smaller from the early‐branching *Pterospora* lineage to the more recently diverged *Monotropa* lineage. This reduction is accompanied by progressively poorer synteny and lineage‐specific loss of *rps23* in *Monotropa*. Phylogenomic analyses of 30 Ericaceae plastomes strongly supported Monotropoideae (bootstrap = 100) and clarified relationships among its 10 sampled genera. Divergence‐time estimation and ancestral‐range reconstruction placed the origin of Clade 1 (Pyroloideae, Arbutoideae and Monotropoideae) in Asia during the late Cretaceous to early Cenozoic. The lineage subsequently dispersed to North America via Beringia, whereas the *Monotropsis* + *Monotropastrum* + *Monotropa* clade later dispersed back to Asia during the late Eocene to early Oligocene. This history gave rise to East Asian endemics, including *Monotropa* humile and *M. callistoma*. These findings illuminate plastome reduction in mycoheterotrophs, resolve relationships within Monotropoideae, and support a complex history of bidirectional intercontinental dispersal.

## Graphical abstract

Phylogenetic Relationships of the Ericaceae Family. ML bootstrap/MP jackknife supported tree from Freudenstein et al. based on (A) nuclear loci, (B) plastid loci, (C) mitochondrial loci, and (D) nuclear+plastid datasets (E). Our combined nuclear + plastid parsimony tree for main groups.

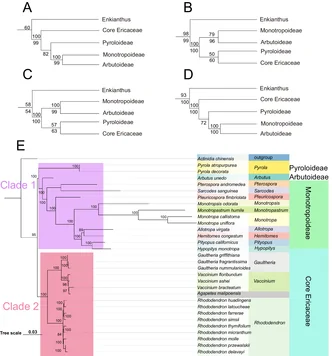

## 1 Introduction

The Ericaceae family comprises perennial herbs, subshrubs, shrubs, or trees and represents a globally distributed family with approximately 3500 species classified into about 125 genera (WFO 2026). It includes numerous economically and ornamentally important plants, such as those from the genera *Vaccinium*, *Rhododendron*, and *Gaultheria*. The infrafamilial classification of Ericaceae has undergone significant changes with advances in plant taxonomy, particularly concerning its closely related subfamilies. These common ancestor lineages include Arbutoideae, Monotropoideae, and Pyroloideae, yet their phylogenetic relationships have not been strictly defined. Traditional taxonomic systems, such as the Cronquist system (1981), treated these groups as a separate family, Pyrolaceae, primarily based on external morphological characteristics like flower structure and herbaceous habit, distinguishing them from the typically woody Ericaceae. However, the APG system (1998) did not recognize this family and instead merged it with Ericaceae. The current mainstream phylogenetic classification, APG III, also does not recognize it as a distinct family, treating it as a subfamily (Pyroloideae) within Ericaceae. This change indicates that molecular systematics strongly supports a close affinity with Ericaceae. In contrast, the Chinese taxonomic system, as represented in the Flora of China, continues to treat Pyrolaceae as a separate family comprising two subfamilies, Pyroloideae and Monotropoideae. Notably, species within Monotropoideae have lost their photosynthetic capacity, resulting in highly simplified morphological features, including chlorophyll‐less shoots and scale‐like leaves. This has long hindered inferences about their ancestry and evolutionary history based on morphology. Therefore, understanding the evolution of fully mycoheterotrophic plants remains one of the most intriguing and challenging questions in plant evolution (Merckx 2013). Based on molecular evidence, Monotropoideae is currently placed as a monophyletic group of achlorophyllous herbs within Ericaceae (The Angiosperm Phylogeny Group 2016; Freudenstein 2016; Li et al. 2021). Initially, Kron et al. (2002) divided Ericaceae into nine subfamilies based on *rbcL*, *matK*, and morphological data, including Enkianthoideae, Pyroloideae, Monotropoideae, Arbutoideae, Cassiopoideae, Ericoideae, Harrimanelloideae, Styphelioideae, and Vaccinioideae. Subsequently, Freudenstein (2016), using a combined analysis of seven genes (three chloroplast and four mitochondrial fragments), found a close relationship between Pyroloideae and a clade comprising Arbutoideae plus Monotropoideae. Later, Rose et al. (2018), based on a combined analysis of 25 genes (13 chloroplast, 9 mitochondrial, and 3 nuclear genes), reported that Arbutoideae is sister to Monotropoideae, while Pyroloideae is sister to all subfamilies excluding Arbutoideae, Enkianthoideae, and Monotropoideae. Most recently, building upon previous findings, Cole et al. (2024) divided Ericales into Monotropoideae, Arbutoideae, Pyrolaceae, and the core Ericaceae. Within Monotropoideae, approximately 12 genera are recognized (Kron et al. 2002), including *Monotropa*, *Monotropsis*, *Hypopitys*, *Pterospora*, *Sarcodes*, *Allotropa*, *Hemitomes*, *Eremotropa*, *Monotropastrum*, *Pityopus*, *Cheilotheca*, and *Pleuricospora*. The majority of these genera are distributed in North America, whereas *Monotropastrum*, *Cheilotheca*, and *Eremotropa* are found in East Asia. Notably, *Hypopitys* and *Monotropa* are the only genera found in both the Old World and the New World (Sorokin et al. 2026). In summary, the phylogenetic relationships within this subfamily and the dispersal routes of its species remain unclear due to a lack of sequencing data for many relevant taxa.

Heterotrophic plants that obtain nutrients from other organisms, either directly from other plants (direct parasites) or through mycorrhizal fungi (mycoheterotrophs) with which they associate, often display a reduction in morphological and genomic features. Due to this shift in lifestyle, the plastomes of mycoheterotrophic plants frequently undergo degradation resulting from relaxed environmental selection pressure. This includes genome size reduction and the loss or pseudogenization of photosynthesis‐related genes (Barrett et al. 2014). Compared to their photosynthetic relatives, almost all mycoheterotrophic plants exhibit reduced plastomes, ranging from minor reductions seen in some “early‐stage” parasitic plants like *Corallorhiza striata* (Barrett and Davis 2012) and *Cuscuta* sp. (Funk et al. 2007) to extreme reductions observed in certain holoparasitic species such as *Sciaphila thaidanica* (Petersen et al. 2018) and *Mitrastemon yamamotoi* (NCBI GenBank accession: NC_080971.1), potentially culminating in the complete loss of the plastid genome, as seen in *Rafflesia lagascae* (Molina et al. 2014). Mycoheterotrophic plants are those that do not perform photosynthesis and rely partially or entirely on mycorrhizal fungi for their carbon supply; such plants commonly exhibit reductions in morphological and genomic features (Bidartondo 2005). Mycoheterotrophic plants can be of three types: fully mycoheterotrophic plants that depend solely on fungal carbon throughout their entire life cycle, initially mycoheterotrophic plants that utilize fungal carbon during their early developmental stages, and partially mycoheterotrophic or mixotrophic plants that combine mycoheterotrophy and autotrophy for carbon acquisition during at least one stage of their life cycle (Li et al. 2022; Baroncelli et al. 2024). Mycoheterotrophy has evolved independently in at least 50 plant lineages, encompassing approximately 400 species (Merckx and Freudenstein 2010). Based on current research, fully mycoheterotrophic plants are found mainly in seven monocot families, namely Orchidaceae, Petrosaviaceae, Triuridaceae, Burmanniaceae, Thismiaceae, Corsiaceae, and Iridaceae, and four eudicot families, namely Polygalaceae, Ericaceae, Gentianaceae, and Rafflesiaceae (Li et al. 2019; Li et al. 2022; Li et al. 2020; Merckx and Freudenstein 2010; Molina et al. 2014; Westwood et al. 2010; Li et al. 2022; Yuan et al. 2025). Among these, Orchidaceae represents the dominant group of mycoheterotrophic plants within monocots, while the Monotropoideae within Ericaceae serves as the representative group within eudicots (Ravin et al. 2016; Westwood et al. 2010). Current research suggests that mycoheterotrophic plants likely evolved from photosynthetic mycorrhizal plants, losing their photosynthetic capacity during evolution and transitioning to dependence on fungi for carbon (Bidartondo 2005). Mycoheterotrophic plants represent an extreme form within the mutualism–parasitism continuum of mycorrhizal symbioses. They occupy unique ecological niches through highly specific fungal associations and provide crucial insights into the evolution and stability of symbiotic systems.

Over the past decade, plastome sequences have been characterized for an increasing number of mycoheterotrophic species across diverse angiosperm lineages. Among monocots, plastome data are now available for multiple representatives of Orchidaceae (e.g., *Corallorhiza*, *Epipogium*, *Neottia*; Barrett et al. 2014; Lam et al. 2018; Schelkunov et al. 2015), as well as for species of Burmanniaceae (e.g., *Burmannia*; Mennes et al. 2015), Triuridaceae (e.g., *Sciaphila*; Petersen et al. 2018), and Thismiaceae (e.g., *Thismia*; Gruenstaeudl 2019). Among eudicots, mycoheterotrophic plastomes have been reported for *Mitrastemon* (Rafflesiaceae; NCBI direct submission), several Gentianaceae species (e.g., *Voyria*; Barrett et al. 2014), and members of Ericaceae. Within Ericaceae, the subfamily Monotropoideae has received particular attention: plastomes have been sequenced for *Monotropa uniflora* (Liu et al. 2020), *M. hypopitys* (Ravin et al. 2016), *Hypopitys monotropa* (as *Monotropa hypopitys* in some studies; Ravin et al. 2016), *Pterospora andromedea* (Braukmann et al. 2017), *Sarcodes sanguinea* (Braukmann et al. 2017), and *Monotropsis odorata* (Braukmann et al. 2017). These plastomes range in size from approximately 26 kb ( *M. uniflora* ) to 41 kb ( *P. andromedea* ), and all exhibit extensive gene loss and structural rearrangements compared to autotrophic relatives, including the loss of the canonical IR/LSC/SSC quadripartite organization in some species (Braukmann et al. 2017; Ravin et al. 2016). Due to their highly simplified morphology, including chlorophyll‐less shoots and scale‐like leaves, inferring the ancestral and evolutionary history of mycoheterotrophic plants based solely on morphology has long been challenging. In this study, we sequenced the complete plastome of *Monotropa callistoma* (Figure 1). This species, belonging to the family Ericaceae, is an achlorophyllous mycoheterotrophic plant that obtains nutrients by parasitizing fungi. Through genome sequencing, we aimed to: (1) characterize the plastome features of *M. callistoma*; (2) investigate its phylogenetic position by comparing it with plastomes of other Monotropoideae species; and (3) conduct comparative analyses of the *M. callistoma* plastome against those of other Monotropoideae members to explore its evolutionary history. These analyses provide a more comprehensive understanding of plastome evolution within the Monotropoideae.

**FIGURE 1:**
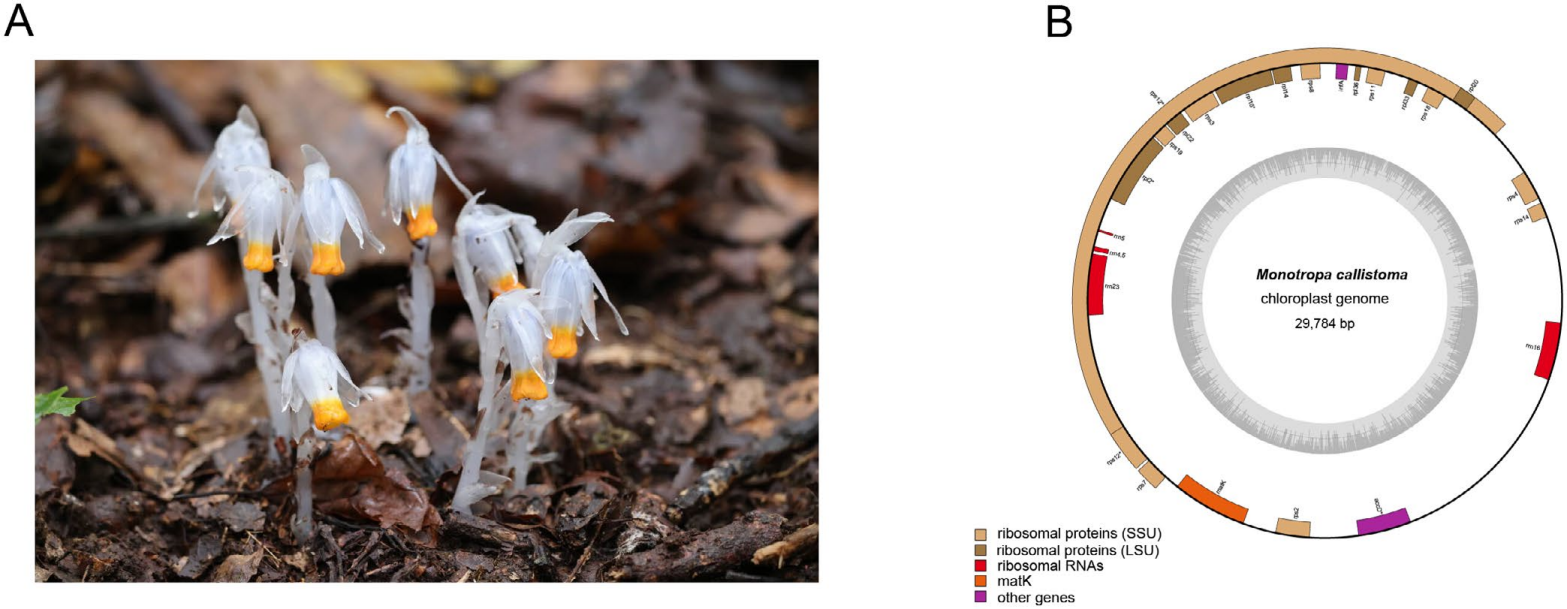
Morphological images of *Monotropa callistoma*. (A) Photograph taken by Dr. Peng Zhan at 110.23° E, 30.06° N. (B) Circular map of the *M. callistoma plastome*. Genes on the outside of the circle are transcribed clockwise, whereas genes on the inside are transcribed counterclockwise. Functional groups are color‐coded: Blue, small‐subunit ribosomal proteins (SSU); cyan, large‐subunit ribosomal proteins (LSU); red, ribosomal RNAs; green, clpP and matK; and gray, other genes. No canonical inverted‐repeat (IR) regions were identified; consequently, the conventional LSC/IR/SSC quadripartite structure is absent.

## 2 Materials and Methods

### 2.1 Sample Collection and Sequencing

Aboveground scale‐leaf tissue of *M. callistoma* (Figure 1) was collected in Shiyan, Hubei Province (110.23° E, 30.06°N). Dr. Tao Yuan of Wuhan University identified the samples, which are preserved in the Herbarium of the School of Life Sciences, Wuhan University (Wuhan, China; Tao Yuan; yuantaosw@163.com). Genomic DNA was extracted using the QIAGEN Genomic Kit, and next‐generation sequencing (NGS) was performed on an Illumina HiSeq 4000 platform at a mean depth of 902.56×. All sequencing procedures were conducted by Beijing Biomarker Biotechnology Co. (Beijing, China).

### 2.2 Plastome De Novo Assembly and Annotation

Raw sequencing reads were processed with BBTools to remove adapter sequences and low‐quality reads (https://sourceforge.net/projects/bbmap/). Clean reads were assembled de novo with GetOrganelle v1.7.5.3 using its default settings (Jin et al. 2020). GetOrganelle uses baiting and iterative mapping to recruit organelle‐associated reads, assembles and filters the graph, and reports possible circular organelle‐genome configurations. The initial assembly was polished with Pilon v1.24 (Walker et al. 2014) using the cleaned short reads to correct potential single‐base errors and short indels. Initial plastome annotation was performed with GeSeq (Tillich et al. 2017), which combines BLAST searches against curated chloroplast databases with tRNA and rRNA prediction. We manually curated the annotation in Geneious Prime (Kearse et al. 2012) to verify start and stop codons and exon–intron boundaries, using *M. odorata* (MF120267.1) as a reference. A gene was considered present when it contained an intact open reading frame (ORF) without internal stop codons and with identifiable start and stop codons. It was considered pseudogenized when a homologous sequence contained internal stop codons, frameshifts or truncations. It was considered absent when no homologous sequence was detected by BLAST against the assembled plastome (E‐value threshold, 1 × 10–5) and the gene was consistently absent from closely related Monotropoideae species (Braukmann et al. 2017; Ravin et al. 2016). All tRNA genes were predicted with tRNAscan‐SE (Chan and Lowe 2019), and plastome maps were generated with OGDRAW (Greiner et al. 2019).

### 2.3 Data Collection and Comparative Analysis of Mycoheterotrophic Plants

We used complete plastome sequences from 31 species representing six families for comparative analyses (Table 1). *Burmannia disticha* (NC_036661.1), which also belongs to Burmanniaceae, was designated as the outgroup. To investigate relationships among mycoheterotrophic plants, we assembled a dataset of 26 protein‐coding genes (PCGs): *accD*, *clpP*, *infA*, *matK*, *rpl2*, *rpl14*, *rpl16*, *rpl20*, *rpl22*, *rpl23*, *rpl32*, *rpl33*, *rpl36*, *rps2*, *rps3*, *rps4*, *rps7*, *rps8*, *rps11*, *rps12*, *rps14*, *rps15*, *rps18*, *rps19*, *ycf1*, and *ycf2*. Sequences were aligned with MAFFT (Katoh et al. 2005), and poorly aligned regions were removed with TrimAl v1.4.1 using the automated1 parameter (Capella‐Gutiérrez et al. 2009). The remaining sequences were concatenated into a supermatrix with FASconCAT‐G v1.0499 (Kück and Longo 2014), yielding a 20,161‐bp alignment. Information on matrix completeness, gene occupancy, and missing‐data patterns is provided in the Data Availability section. Maximum‐likelihood (ML) analyses were performed in IQ‐TREE (Nguyen et al. 2015). ModelFinder selected the substitution model and partitioning strategy for each dataset using the Bayesian information criterion (Kalyaanamoorthy et al. 2017). Analyses used the GTR + F + I + I + R3 model, SH‐aLRT tests, and 5000 bootstrap replicates. The resulting trees were visualized with the ChiPlot online tool (Xie et al. 2023).

**TABLE 1:** Summary of plastome information for 30 mycoheterotrophic plants.

Species	Length (bp)	GC content (%)	Family	Accession number
*Hypopitys monotropa*	35,336	34.3	Ericaceae	NC_029704.1
*Monotropa uniflora*	26,913	27.1	Ericaceae	MT491729.1
*Monotropa callistoma*	29,784	30.8	Ericaceae	This study
*Monotropsis odorata*	32,752	31.2	Ericaceae	MF120267.1
*Allotropa virgata*	35,983	33.1	Ericaceae	MF120261.1
*Hemitomes congestum*	35,569	33.8	Ericaceae	MF120263.1
*Pityopus californicus*	35,926	34.4	Ericaceae	MF120268.1
*Pterospora andromedea*	40,420	33.8	Ericaceae	MF120270.1
*Monotropastrum humile*	30,709	30.8	Ericaceae	PQ304570.1
*Mitrastemon yamamotoi*	18,252	25.1	Rafflesiaceae	NC_080971.1
*Burmannia nepalensis*	98,706	34.7	Burmanniaceae	NC_036670.1
*Burmannia oblonga*	39,386	33.2	Burmanniaceae	NC_036664.1
*Burmannia championii*	85,868	35.6	Burmanniaceae	NC_036663.1
*Burmannia cryptopetala*	107,771	36.5	Burmanniaceae	NC_036662.1
*Lecanorchis japonica*	70,498	30.4	Orchidaceae	NC_046799.1
*Lecanorchis kiusiana*	74,084	30	Orchidaceae	NC_046798.1
*Gastrodia pubilabiata*	30,698	25	Orchidaceae	NC_082115.1
*Gastrodia uraiensis*	30,746	25.5	Orchidaceae	NC_066067.1
*Gastrodia shimizuana*	30,019	25.8	Orchidaceae	NC_066065.1
*Gastrodia peichatieniana*	29,696	25.2	Orchidaceae	NC_066064.1
*Gastrodia javanica*	31,896	26.9	Orchidaceae	NC_066063.1
*Gastrodia crispa*	30,582	25.4	Orchidaceae	NC_066062.1
*Gastrodia flexistyla*	30,797	25.3	Orchidaceae	NC_066061.1
*Gastrodia angusta*	36,812	25.9	Orchidaceae	NC_066060.1
*Gastrodia menghaiensis*	30,158	24.9	Orchidaceae	NC_066069.1
*Gastrodia elata*	35,304	26.8	Orchidaceae	NC_037409.1
*Gastrodia longistyla*	30,464	24.8	Orchidaceae	NC_063317.1
*Sciaphila thaidanica*	12,780	30.5	Triuridaceae	MG757197.1
*Sciaphila densiflora*	21,485	39.9	Triuridaceae	NC_027659.1
*Exacum paucisquama*	44,028	36.2	Gentianaceae	—

### 2.4 Data Collection and Phylogenetic Analysis of Ericaceae

To investigate phylogenetic relationships within Monotropoideae, we downloaded complete plastomes for 28 species representing 15 Ericaceae genera and added published gene sequences for *P. fimbriolata* and *S. sanguinea* (Table S1). *Actinidia chinensis* (Actinidiaceae) was used as the outgroup. PCGs were extracted with PhyloSuite (Zhang et al. 2020), and the concatenated alignment was 47,746 bp long. Gene‐occupancy and missing‐data statistics are provided in the Data Availability section. Sequence processing and phylogenetic analyses followed the workflow described above and in Zhang et al. (2025a, 2025b), Zhang et al. (2024), and Nawae et al. (2026). For this dataset, ModelFinder selected a single unpartitioned scheme with the GTR + F + I + I + R4 model under the Bayesian information criterion. ML analyses were conducted in IQ‐TREE with SH‐aLRT tests and 5000 bootstrap replicates.

Divergence times for Ericaceae were estimated from seven shared plastome PCGs. Four fossil calibrations based on published palaeobotanical evidence from TimeTree (https://timetree.org/home) were selected (Table S2). The MCMC analysis ran for 2 million generations, sampling every 1000 generations; the first 20% of samples were discarded as burn‐in. Convergence was assessed with Tracer v1.7.2 (Rambaut et al. 2018), and all effective sample sizes (ESSs) exceeded 200. Divergence‐time estimates were visualized with ChiPlot (Xie et al. 2023), with node bars showing 95% highest posterior density (HPD) intervals.

### 2.5 Plastome Alignment of Monotropoideae

The plastomes of Monotropoideae were aligned and visualized using the Mauve module in Geneious Prime (Kearse et al. 2012). The results of this whole‐genome alignment were graphically overlaid onto the phylogenetic topology inferred from the alignment of 26 trimmed plastome PCGs.

### 2.6 Biogeographical Reconstruction

To reconstruct Ericaceae biogeographic history, we estimated ancestral ranges with RASP (Yu et al. 2020). A time‐calibrated tree was obtained with the MCMCTree module in PAML (Yang 2007) from seven plastid PCGs (*rps4*, *rps7*, *rps8*, *rps11*, *rpl33*, *rpl22*, and *rpl14*). These genes were selected because they are present and alignable across all 28 Ericaceae species included in this study, whereas other PCGs show variable loss or pseudogenization in the reduced Monotropoideae plastomes. They have also been used in previous phylogenetic and biogeographic studies of Ericaceae (Freudenstein 2016; Rose et al. 2018; Gao et al. 2023). Four fossil calibrations based on published palaeobotanical evidence from TimeTree (https://timetree.org/home) were selected (Table S2).

We used three RASP methods to estimate ancestral‐range probabilities: the statistical BioGeoBEARS package (S‐BGB; Matzke 2013, 2014), statistical dispersal–vicariance analysis (S‐DIVA), and binary Bayesian MCMC (BBM). For S‐BGB, we tested all six BioGeoBEARS models (DEC, DEC + J, DIVALIKE, DIVALIKE+J, BAYAREALIKE + J). Model selection used AICc weights, and DIVALIKE + J was selected as the best‐fitting model (AICc weight = 9.50E‐01). The principal biogeographic conclusions were consistent across all six models. For BBM, we used the F81 + model and ran four MCMC chains for 50,000,000 generations, sampling every 5000 generations and discarding the first 25% as burn‐in. Using GBIF (GBIF 2025), the Flora of China (2025), and collection records, we defined four distribution areas: A, Asia; B, Europe; C, North America; and D, South America (Table S3). The maximum number of ancestral areas at any node was set to three.

## 3 Results

### 3.1 General Characteristics of *M. callistoma* Plastome

We report the plastome of M. *callistoma* for the first time. *The* assembly comprised a single circular molecule. The plastome is 29,784 bp long and has a GC content of 30.8% (Figure 1). It lacks the canonical quadripartite structure, which consists of two inverted repeats separating the large and small single‐copy regions. No clear IR boundaries were identified in the assembly or annotation (Figure 1). The plastome is intermediate in size between those of *M. uniflora* (26,913 bp) and *Hypopitys monotropa* (35,336 bp), and its GC content is also intermediate (Liu et al. 2020; Ravin et al. 2016). We identified 40 genes, including 20 protein‐coding genes (PCGs), 16 tRNA genes and four rRNA genes (Table 2). The PCGs include ten genes encoding small‐subunit ribosomal proteins, seven encoding large‐subunit ribosomal proteins, and three additional genes (*infA*, *matK*, and *accD*). Four PCGs (*accD*, *rpl2*, *rpl16*, and *rps12*) contain one intron.

**TABLE 2:** Genes present in assembled plastome of *Monotropa callistoma*.

Category	Gene group	Gene name
Photosynthesis	Subunits of photosystem I	—
	Subunits of photosystem II	—
	Subunits of NADH dehydrogenase	—
	Subunits of cytochrome b/f complex	—
	Subunits of ATP synthase	—
	Large subunit of rubisco	—
	Subunits photochlorophyllide reductase	—
Self‐replication	Proteins of large ribosomal subunit	*rpl14*,rpl16*,rpl2*,*rpl20*,*rpl22*,*rpl33*,*rpl36*
	Proteins of small ribosomal subunit	*rps11*,rps12*,*rps14*,*rps18*,*rps19*,*rps2*,*rps3*,*rps4*,*rps7*,*rps8*
	Subunits of RNA polymerase	—
	Ribosomal RNAs	*rrn16*,*rrn23*,*rrn4*.*5*,*rrn5*
	Transfer RNAs	*trnC‐GCA*,*trnD‐GUC*,*trnE‐UUC*,*trnF‐GAA*,*trnfM‐CAU*,*trnH‐GUG*,*trnI‐CAU*,*trnN‐GUU*,*trnP‐UGG*,*trnQ‐UUG*,*trnS‐GCU*,*trnS‐UGA*,trnV‐GAC*(2),*trnW‐CCA*,*trnY‐GUA*
Other genes	Maturase	*matK*
	Protease	—
	Envelope membrane protein	—
	Acetyl‐CoA carboxylase	accD*
	C‐type cytochrome synthesis gene	—
	Translation initiation factor	*infA*
	Other	—
Genes of unknown function	Conserved hypothetical chloroplast ORF	—

Abbreviations: Gene(2), number of copies of multi‐copy genes; Gene*, genes with one intron.

### 3.2 Comparative Analysis of Plastomes in Mycoheterotrophic Plants

We compiled published plastomes from 30 mycoheterotrophic species. The plastomes of *Sciaphila thaidanica*, *S. densiflora* (Triuridaceae) and *Mitrastemon yamamotoi* (Rafflesiaceae) were extremely contracted, with lengths of approximately 20 kb (Figure 2). GC content showed a weak but statistically significant positive association with genome size (*R* = 0.1862, *p* < 0.05; Figure 2), indicating that genome size explains only a limited proportion of GC‐content variation. Similar patterns have been reported in other holoparasitic plants, including *Hydnora, Prosopanche*, and *Lactoris* (Jost et al. 2022). Within Monotropoideae, *P. andromedea* had the largest plastome, whereas *M. uniflora*, a more recently diverged lineage, had the smallest. Synteny was poor among Monotropoideae species, particularly in *Hypopitys, Monotropsis, Monotropastrum*, and *Monotropa* (Figure S1). Synteny was relatively well conserved within *Monotropa*, but fragment loss increased in more recently diverged lineages, resulting in progressively poorer intergeneric synteny.

**FIGURE 2:**
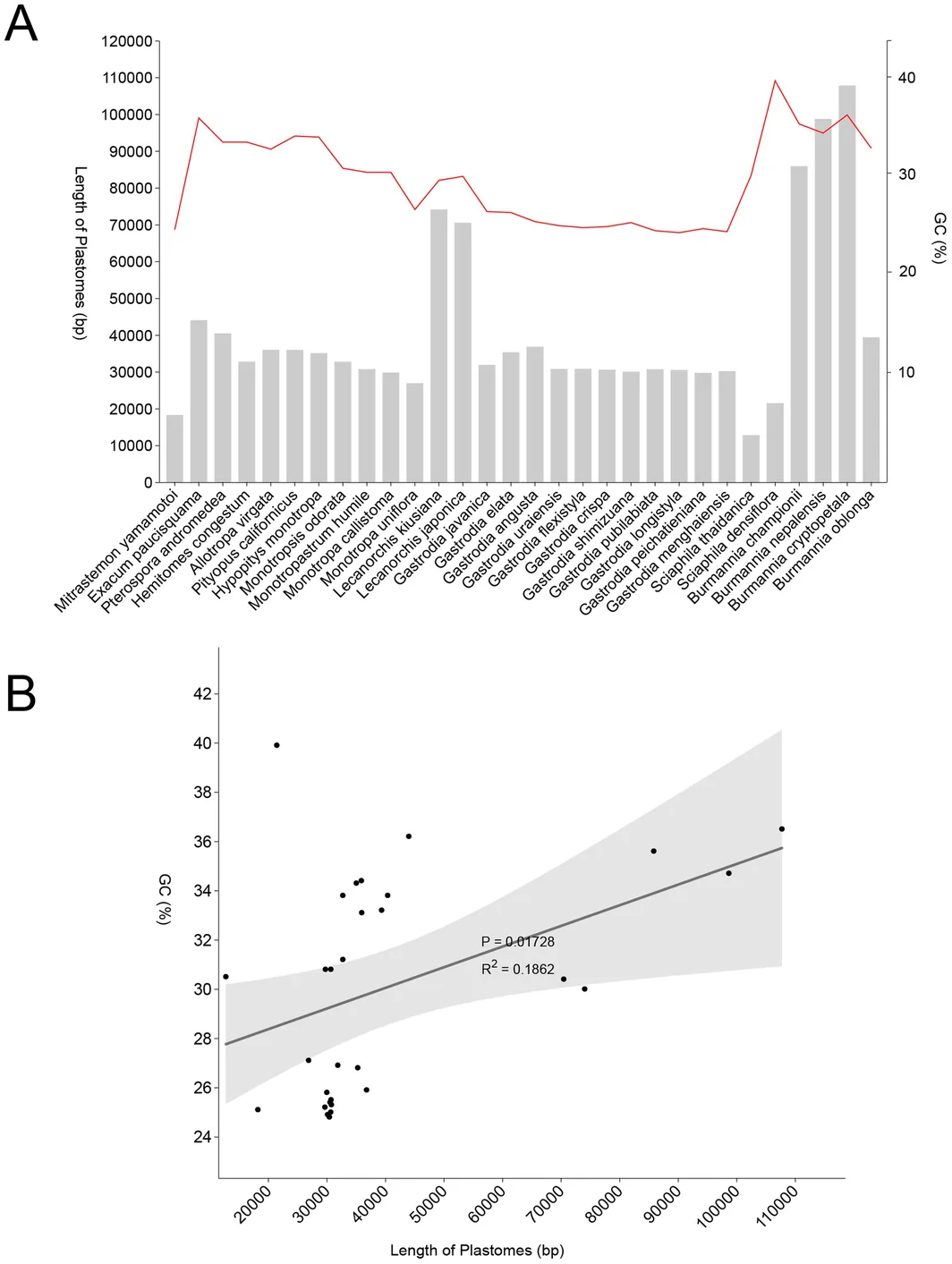
Genomic characteristics of plastomes. (A) Plastome length and GC content for 30 mycoheterotrophic species. (B) Positive association between GC content and genome length.

We reconstructed a phylogenetic tree for these relatively distantly related species, which formed monocot and eudicot clades (Figure 3). The *accD* gene was retained in all species, whereas *ccsA*, *cemA*, *ndh*, *psa*, *psb*, *rpo*, *ycf3*, and *ycf4* were absent from all sampled taxa. Gene loss was more extensive in eudicots than in monocots; for example, *atp*, *ycf1*, and *ycf2* were frequently lost in eudicot lineages. Ancestral‐state reconstruction indicated convergent losses of *ycf1* and *ycf2* among eudicots. All sampled *Monotropa* species lacked *rps23*, suggesting a lineage‐specific loss within the genus.

**FIGURE 3:**
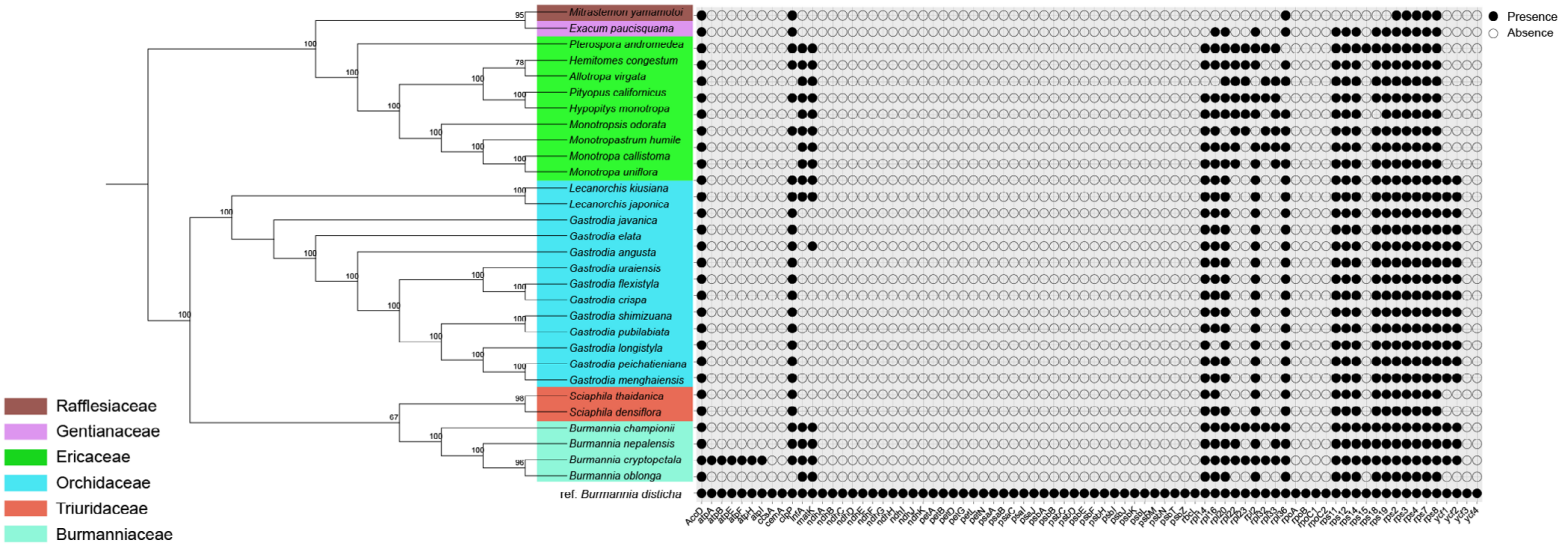
Phylogenetic relationships and plastid gene‐loss patterns in mycoheterotrophic plants. Left: Maximum‐likelihood phylogeny inferred from 26 protein‐coding genes (PCGs), with bootstrap support values indicated at the nodes. Right: Patterns of plastid gene retention and loss across 30 mycoheterotrophic species. Filled circles indicate gene retention, whereas open circles indicate gene loss.

### 3.3 Phylogenetic Analysis of Ericaceae

The ML tree based on plastid PCGs divided Ericaceae into two major clades, Clades 1 and 2 (Figure 4). This result is consistent with the combined nuclear and plastid analysis of Freudenstein (2016). Clade 1 comprised Pyroloideae, Arbutoideae, and Monotropoideae. Pyroloideae was the earliest‐branching lineage in Clade 1, whereas Monotropoideae was the most recently diverged. Our sampling included 10 Monotropoideae genera and provided strong resolution of their relationships (bootstrap = 100). *Pterospora* and *Sarcodes* occupied successive early‐branching positions, followed by *Pleuricospora*. The *Monotropsis* + *Monotropastrum* + *Monotropa* clade was sister to the *Allotropa* + *Hemitomes* + *Pityopus* + *Hypopitys* clade. Clade 2 comprised the core Ericaceae and was subdivided into Vaccinioideae and Ericoideae (Figure 4).

**FIGURE 4:**
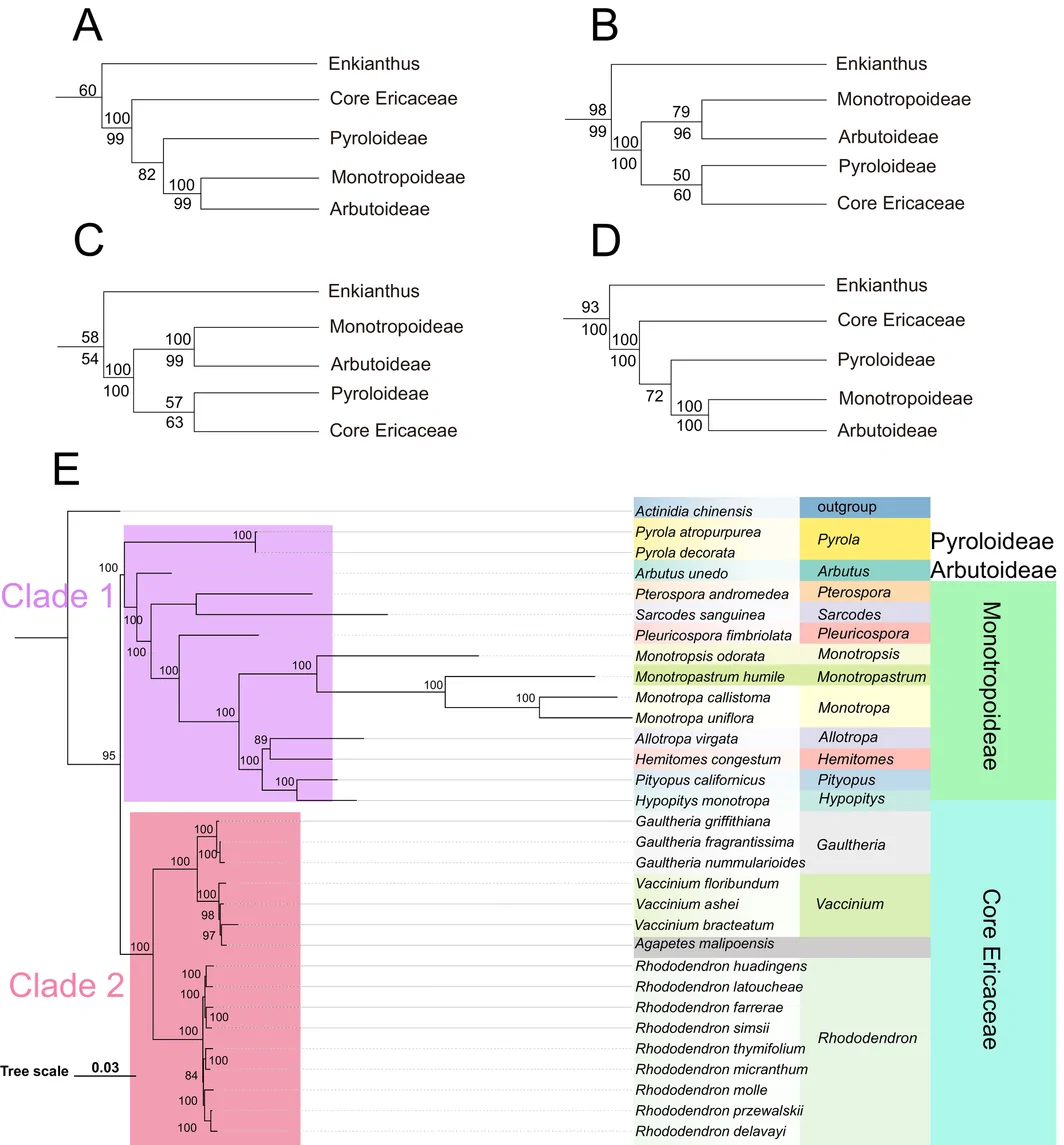
Phylogenetic relationships in Ericaceae. ML bootstrap and MP jackknife‐supported trees from Freudenstein and Senyo (2008) based on (A) nuclear loci, (B) plastid loci, (C) mitochondrial loci, and (D) nuclear + plastid datasets. (E) Species tree inferred from combined nuclear and plastid data. Right‐hand bars and species names are color‐coded by subfamily or clade: Yellow, Pyroloideae; blue, Arbutoideae; green, Monotropoideae; and light blue, core Ericaceae. *Actinidia chinensis* was used as the outgroup. Node values denote bootstrap percentages.

### 3.4 Divergence Time and Ancestral Range Dispersal

Fossil‐calibrated divergence‐time estimates placed the split between Pyroloideae and the remaining subfamilies at approximately 67.77 Ma (Figure 5). Arbutoideae and Monotropoideae diverged at approximately 66.57 Ma, and diversification within Monotropoideae began about 63.35 Ma. Within the subfamily, *Sarcodes* and *Pterospora* diverged approximately 45.79 Ma. *Monotropastrum* and *Monotropa* diverged about 26.74 Ma, after their lineage split from *Monotropsis* approximately 39.73 Ma. *Pityopus* and *Hypopitys* diverged approximately 20.19 Ma, whereas *Hemitomes* and *Allotropa* diverged approximately 32.55 Ma. The clade containing these two pairs diverged approximately 36.71 Ma. Vaccinioideae and Ericoideae diverged approximately 26.59 Ma, followed by rapid radiation in *Rhododendron* and *Vaccinium* around 1–2 Ma. Ancestral‐range estimation placed the most recent common ancestor of Clade 1 in Asia during the late Cretaceous to early Cenozoic, followed by dispersal to North America (Figure 6). The *Monotropsis* + *Monotropastrum* + *Monotropa* lineage subsequently appears to have dispersed back to Asia during the late Eocene to early Oligocene (approximately 39.70 Ma), giving rise to East Asian endemics such as *M. humile* and *M. callistoma*.

**FIGURE 5:**
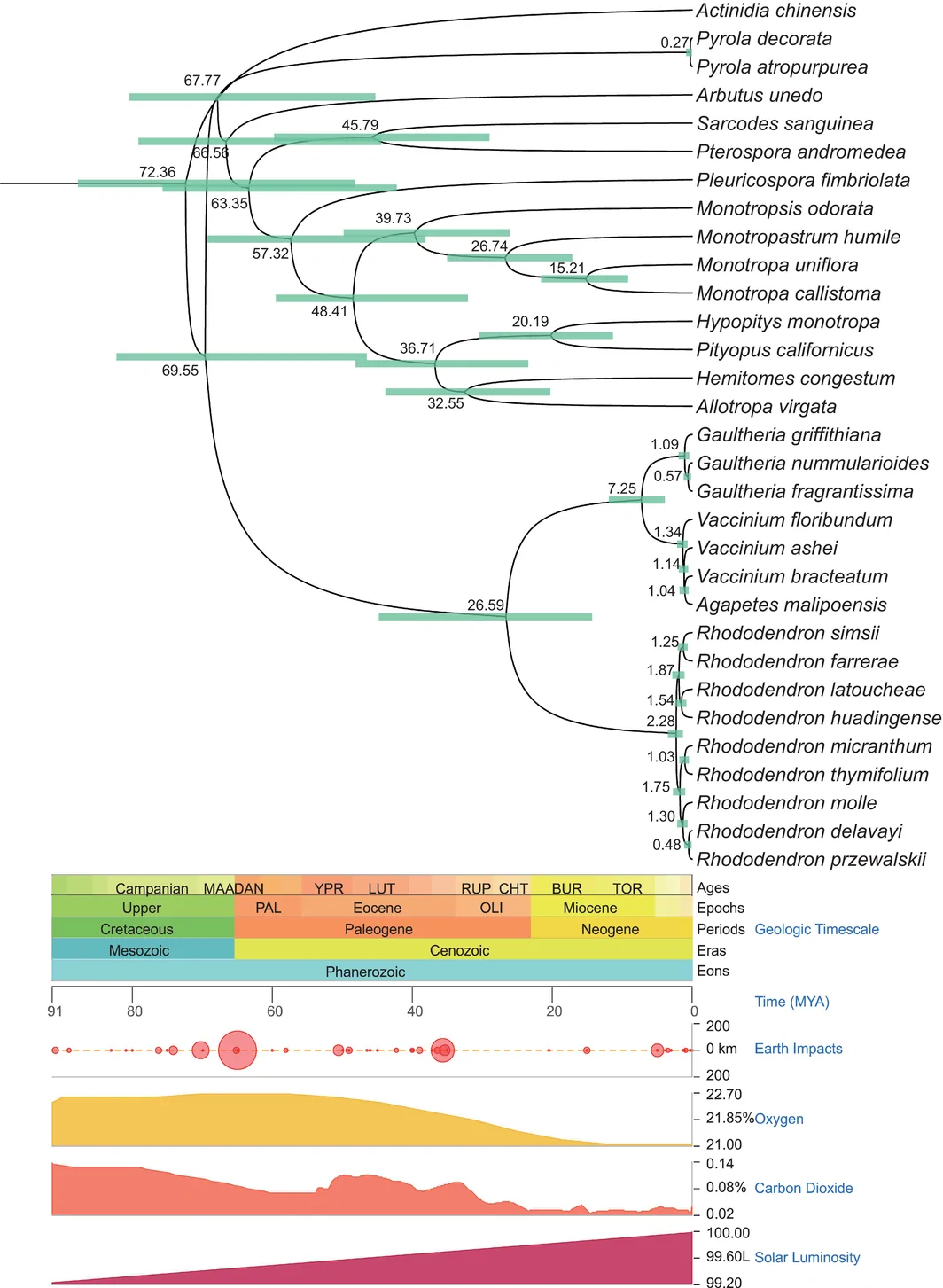
Chronogram showing divergence times among 31 plants. Green bars represent 95% highest posterior density intervals, and black numbers above branches indicate divergence times.

**FIGURE 6:**
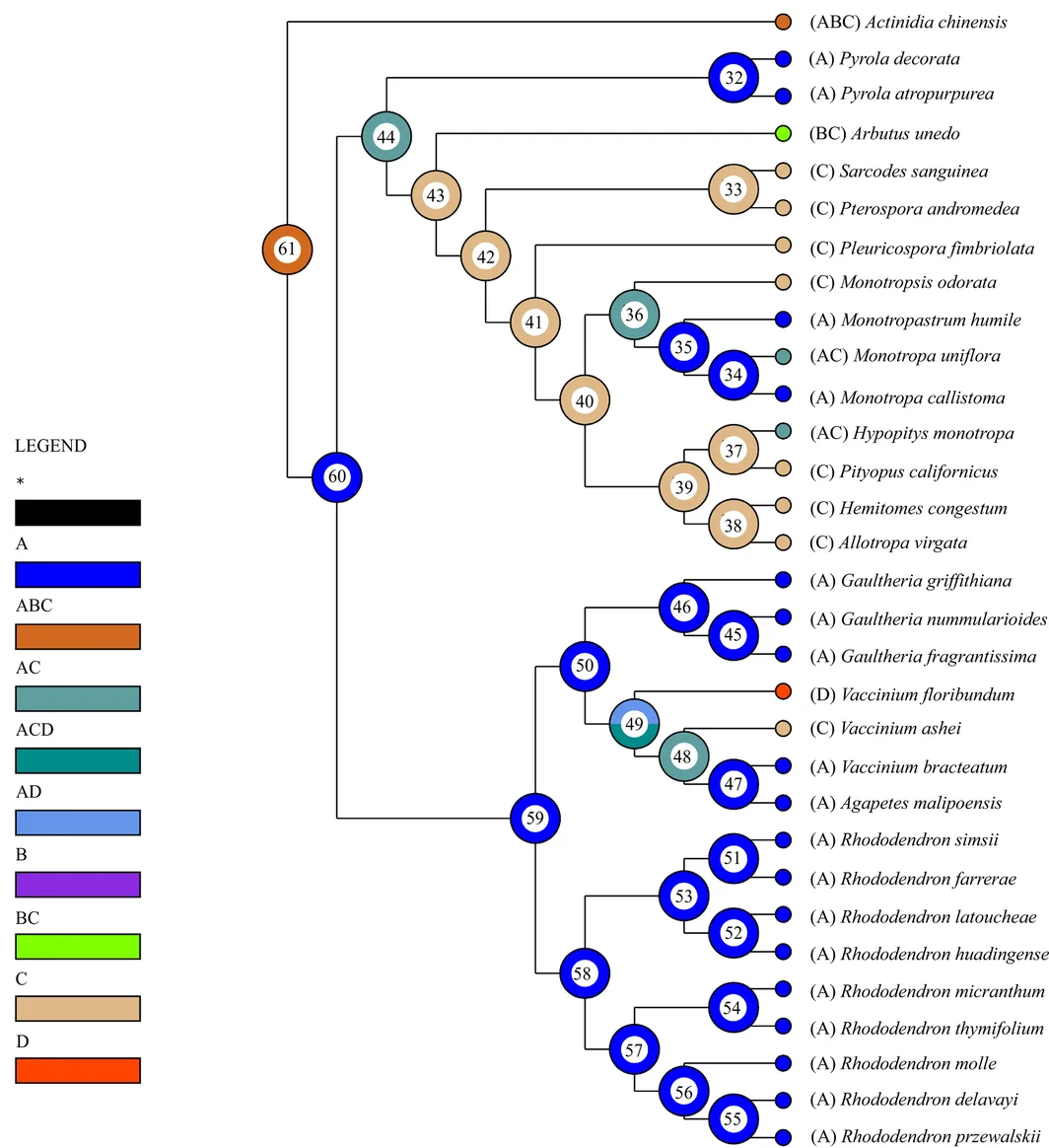
Ancestral‐range estimation for Ericaceae based on the DIVALIKE+J model implemented in RASP (Yu et al. 2020). Tip colors indicate the current distribution of each taxon. Pie charts at internal nodes show marginal probabilities for alternative ancestral areas: A, Asia; B, Europe; C, North America; and D, South America. Only the most likely ancestral ranges are shown; probabilities below 5% are pooled in gray.

## 4 Discussion

### 4.1 Phylogenetic Relationships Within Monotropoideae

The plastome‐based phylogeny provides a well‐resolved framework for relationships within Monotropoideae. *Pterospora* and *Sarcodes* formed successive early‐branching lineages, followed by *Pleuricospora*. This topology is broadly consistent with the ITS analysis of Bidartondo and Bruns (2001), while providing stronger resolution among the remaining genera. The *Monotropsis* + *Monotropastrum* + *Monotropa* clade was sister to the *Allotropa* + *Hemitomes* + *Pityopus* + *Hypopitys* clade, with strong bootstrap support (100%). The inferred topology, ((*Pterospora*, *Sarcodes*), ((*Monotropsis*, (*Monotropa*, *Monotropastrum*)), ((*Allotropa*, *Hemitomes*), (*Pityopus*, *Hypopitys*)))), is consistent with Braukmann et al. (2017) and differs from arrangements based on partial 26S sequences (Neyland and Hennigan 2004; Neyland 2005).

The supported sister relationships between *Allotropa* and *Hemitomes* and between *Pityopus* and *Hypopitys* further clarify the terminal group. The *Monotropa* + *Monotropastrum* + *Monotropsis* clade had exceptionally long branches, indicating elevated substitution rates in their plastid genes. This acceleration may reflect relaxed selection following the loss of photosynthetic function, consistent with the finding that plastid genes in *Monotropa* evolve approximately 13 times faster than nuclear genes (Freudenstein and Broe 2024). These lineages also have smaller plastomes than early‐branching members of the subfamily, consistent with more extensive genome erosion during prolonged mycoheterotrophic evolution. Together, relaxed selection, elevated substitution rates, and genome reduction are consistent with progressive plastome degradation during the evolution of obligate mycoheterotrophy in Monotropoideae.

Our analysis included 10 of the 12 recognized Monotropoideae genera because samples of the Asian endemics *Cheilotheca* and *Eremotropa* were unavailable. Their phylogenetic positions therefore remain unresolved. Plastome data from these genera will be needed to complete the subfamily‐level framework and refine inferences about character evolution.

### 4.2 Ancestral Range Dispersal

We reconstructed ancestral distributions and dispersal history in Ericaceae. The results support an Asian origin for the most recent common ancestor of Clade 1 during the late Cretaceous to early Cenozoic, followed by dispersal to North and South America. The Bering Land Bridge was a potential dispersal corridor between Asia and North America from the latest Cretaceous to the early Paleocene (approximately 65.5 Ma) (Brikiatis 2014). Molecular and biogeographic analyses of *Cryptocercus* indicate that the genus occupied both continents by the late Cretaceous and support dispersal from Asia to the Americas via Beringia (Che et al. 2016). A study of *Tetragnatha* similarly inferred movement from eastern Asia to western North America through Beringia (Zhang, Xiang, et al. 2025). Across plants, fungi, and animals, dispersal from Asia to North America was more frequent during the Cenozoic, whereas North America‐to‐Asia dispersal peaked during the late Eocene (approximately 40–34 Ma) (Jiang et al. 2019). Fossil evidence also indicates that the Asian rodent *Pappocricetodon* dispersed to North America at least twice, once during the middle Eocene and again near the end of the Eocene (Tong 1992).

The ancestor of the *Monotropsis* + *Monotropastrum* + *Monotropa* clade likely dispersed back to Asia during the late Eocene to early Oligocene (approximately 39.70 Ma), giving rise to endemics such as *M. humile* and *M. callistoma*. This inference is consistent with a land connection between Asia and North America. The Clade 1 ancestor probably moved from East Asia to North America through Beringia during the Paleocene, when the region was warmer and more humid than today. Beringia supported a continuous belt of deciduous broadleaf forest and some tropical evergreen forest during this period (Wolfe 1975, 1978; Huang et al. 2024), facilitating dispersal by warm‐temperate taxa (Tiffney 1985). Overall, the results and fossil evidence support an Asian origin for Clade 1, followed by dispersal to North America and a later return to Asia by the *Monotropsis* + *Monotropastrum* + *Monotropa* lineage. Beringia may therefore have played an important role in Ericaceae biogeography.

### 4.3 Convergent Loss of Photosynthesis‐Related Genes

We observed that among the 30 currently published plastomes of mycoheterotrophic plants, with the sole exception of *B. cryptopetala*, all other species have completely lost the photosynthesis‐related *atp*, *ndh*, *pet*, *psa*, *psb*, and *rpo* genes, as well as *rbcL*, *ycf3*, and *ycf4*. The loss of photosynthetic capacity is correlated with a significant reduction in plastid coding capacity, consistent with the evolutionary trend of plastome reduction (Martin et al. 1998). The universal absence of two genes not directly involved in photosynthesis (*ycf3* and *ycf4*) across all species is expected, given the near‐complete degradation of genes encoding both photosystem complexes in these plants (Figure 2). The *rbcL* is completely lost in all mycoheterotrophs examined but is retained in some parasitic lineages, such as *Cuscuta* (McNeal et al. 2007), *Aneura* (Wickett et al. 2008), *Petrosavia* (Cameron et al. 2003), and *Pleuricospora fimbriolata* (Braukmann and Stefanović 2012). Although the differential retention of rbcL *between* mycoheterotrophic and parasitic lineages remains unexplained, it may be related to differences in their hosts.

The *ndhA‐K* genes encode components of the thylakoid NADH dehydrogenase complex, primarily involved in cyclic electron transport (Martín and Sabater 2010). We found that ndh genes were completely absent in all species, consistent with previous findings in *Oncidium*, *Corallorhiza*, *Phalaenopsis*, *Neottia*, and *Rhizanthella* (Barrett and Davis 2012). Given that mycoheterotrophic plants do not rely on photosynthesis, the loss of *ndh* genes is expected. However, it is interesting to note that *ndh* genes have also been lost in some photosynthetic plant plastomes, including the order Gnetales (Braukmann et al. 2009) and Geraniaceae (Chris Blazier et al. 2011), and have become pseudogenes in certain lineages of the monocot order Alismatales (Iles et al. 2013). While the loss of *ndh* genes from autotrophic plant plastomes is not fully understood, the existence of a second form might explain their multiple losses (Wicke et al. 2011). In summary, mycoheterotrophic plants (excluding photosynthetic orchids) maintain full fungal dependence throughout their life cycle, and the convergent loss of *ndh* genes in mycoheterotrophic plants is consistent with convergent evolution in the host‐fungus relationship. The observed patterns are consistent with a scenario in which the progressive shift from autotrophy toward full mycoheterotrophy is accompanied by the convergent loss of photosynthesis‐related genes across independent lineages. We interpret these patterns as evidence of convergent evolution, similar gene‐loss outcomes arising independently in distantly related lineages under similar selective pressures. However, we acknowledge that distinguishing true convergence from parallel evolution due to shared ancestry or similar genomic architectures would require more rigorous comparative frameworks and broader taxon sampling.

The *atp* genes were lost in all species except *B. cryptopetala*. This situation presents a notable exception, as *B. cryptopetala* and a few other taxa, such as the non‐angiosperm non‐photosynthetic plant *Aneura mirabilis* (not included in this study), differ from the majority of fully mycoheterotrophic plants that have completely lost *atp* genes from their plastomes (Wickett et al. 2008). Further research has revealed that the retention of *atp* genes is closely associated with plant nutritional strategies: these genes are often retained in mixotrophic plants (e.g., *Corallorhiza*) or parasitic plants (e.g., *Cuscuta*) (Barrett and Davis 2012).

Therefore, current evidence suggests that the systematic loss of *atp* genes is correlated with the transition from partial to full obligate mycoheterotrophy, and may serve as a molecular signature of extensive functional remodeling of the plastome. However, we acknowledge that functional validation and broader taxon sampling would be required to confirm the generality of this pattern. During the degradation process of plant plastid genomes, the *clpP* gene, encoding the core catalytic subunit of the Clp protease, is generally considered one of the last to be lost in the terminal stages of plastid degradation. Consequently, *clpP* is retained in the plastids of many parasitic and hemiparasitic plants (Hadariová et al. 2018). However, the retention pattern of *clpP* in fully mycoheterotrophic plants shows significant variation. For instance, it is present in the highly reduced plastomes of some mycoheterotrophic orchids (e.g., *Corallorhiza*), but has been completely lost in evolutionarily more derived genera such as *Monotropa*.

This phenomenon is highly consistent with the extremely long branch lengths observed for *Monotropa* in phylogenetic trees. Long branches typically suggest that these lineages have experienced accelerated molecular evolutionary rates. Combined with their extremely heterotrophic lifestyle involving complete loss of photosynthesis, the most parsimonious explanation is that the entire plastome of *Monotropa* has undergone stronger relaxed selection. The complete abandonment of photosynthetic function has released numerous plastid genes, including *clpP*, from purifying selection, thereby accelerating their progression through pseudogenization to eventual loss. The complete loss of *clpP* in *Monotropa*, in combination with the long branch lengths observed for this genus, is consistent with a scenario in which these lineages have experienced exceptionally strong relaxed selection across their plastomes. While this pattern may represent an advanced stage of plastid degradation within Monotropoideae, we caution that functional evidence and direct selection analyses would be required to confirm a causal link between *clpP* loss and the transition to full mycoheterotrophy. We therefore interpret *clpP* loss as a potential molecular marker associated with extensive plastome reduction in this lineage, rather than as a definitive indicator of an ‘ultimate’ evolutionary transition.

The extensive gene loss observed in plastomes of fully mycoheterotrophic organisms conforms to the final stage of the plastome degradation model proposed by Barrett and Davis (2012). Based on the observed patterns across the 30 mycoheterotrophic species examined, we tentatively propose a testable hypothesis regarding the relative order of plastid gene loss: *ndh* → *psa*/*psb*, *pet*, *rbcL*, *ycf3*/*4* → *rpo* → *atp* → *rpl*, *rps*, *rrn*, *trn*, *accD*, *clpP*, *matK*, *ycf1*/*2*. However, we emphasize that this proposed order is inferred from static comparisons across independent lineages and does not directly reconstruct a universal temporal sequence of loss. Testing this hypothesis would require direct selection analyses, functional studies, and broader taxon sampling across multiple independent origins of mycoheterotrophy. Furthermore, the degree of dependence on fungal hosts may correlate with the extent of gene loss, although we acknowledge that this correlation is not rigorously tested by our current dataset and remains a hypothesis for future investigation.

Accordingly, we hypothesize that the degree of a plant's absolute dependence on fungal hosts is positively correlated with the breadth and depth of plastid gene loss. Lineages with higher dependence and more specialized lifestyles, such as *Monotropa*, experience more complete relaxation of selective constraints on their plastomes, resulting in more comprehensive gene loss until only a minimal set of genes essential for basic plastid maintenance remains. As Bidartondo (2005) describes, the study of mycorrhizal cheaters like *A. mirabilis* remains a field in its “infancy”. In conclusion, the systematic loss of photosynthesis‐related genes in fully mycoheterotrophic plants illustrates the evolutionary trajectory of plastids following the release of functional constraints: it does not follow random, lineage‐specific paths, but converges toward extreme genomic simplification driven by strong relaxed selection. This reveals a profound evolutionary principle: when an organelle's core function is “outsourced” by its host, the decay and loss of its genetic system is a frequently observed outcome under relaxed selection.

### 4.4 Convergent Conservation of Housekeeping Genes

The loss of photosynthesis‐related genes in angiosperm plastids has occurred multiple times independently. Similar to the degree of plastid degradation observed in mycoheterotrophs, analogous phenomena can be found in unrelated fully mycoheterotrophic plant lineages, including some parasitic plants (Whitton et al. 2008). Another example of convergent plastome reduction in holoparasitic plants is evident in the completely parasitic family Cytinaceae. The plastid genomes of five *Cytinus* species and three *Bdallophytum* species are comparable in size and gene content to those of Monotropoideae, retaining many housekeeping genes such as *rps*, *rpl*, and *accD* (Jost et al. 2022). Housekeeping genes exhibit remarkable similarity across mycoheterotrophic organisms. The *rps* and *rpl* genes, encoding ribosomal small and large subunits, along with other “housekeeping genes” (e.g., *accD*, *clpP*, *infA*, *matK*, *ycf1*, *ycf2*), are retained in most species (Figure 2). Notably, the *accD* gene is retained in the plastomes of all mycoheterotrophic species examined.

In the plastomes of fully mycoheterotrophic plants, the retention patterns of the two largest genes, *ycf1* and *ycf2*, exhibit striking lineage‐specificity. Unlike photosynthesis‐related genes, which are consistently lost across nearly all groups, *ycf1* and *ycf2* are retained only in certain species of Orchidaceae and Burmanniaceae, but have been completely lost in other fully mycoheterotrophic lineages. This unique retention pattern may be related to their complex and not yet fully understood functions. Although *ycf1* and *ycf2* have been shown to be essential for plastid survival and function in autotrophic plants like tobacco (Drescher et al. 2000), studies have also found they can be lost or rendered non‐functional in some lineages, such as Poaceae (Downie et al. 1994), indicating lineage‐dependent differences in their functional importance. Notably, in mycoheterotrophic orchids that retain both genes, they show signs of significantly relaxed selective constraint (elevated dN/dS ratios) (Barrett and Davis 2012). This suggests that even when retained, these genes have largely escaped purifying selection and may be on a trajectory toward functional degradation or pseudogenization. Therefore, the loss or retention of *ycf1* and *ycf2* cannot be simply interpreted as indicators of the “stage” of plastid degradation. Instead, it likely reflects fundamental differences in the last remaining functional constraints imposed on the plastid among different lineages during adaptation to full mycoheterotrophy. Orchidaceae and Burmanniaceae may retain some unrecognized fundamental metabolic requirements dependent on *ycf1*/*ycf2* function, whereas other lineages, such as Monotropoideae, have apparently relieved these constraints, resulting in extreme simplification of their plastid genomes. The functional significance of this lineage‐specific retention remains unclear and warrants further investigation.

Maturase K (*matK*) encodes a maturase‐like protein, MatK, whose function is broadly considered to be post‐transcriptional processing of group IIA intron‐containing genes (Zoschke et al. 2010). Previous research indicated extensive matK loss in the subtribe Corallorhizinae, with the highest degree of loss in *Corallorhiza*, followed by related genera *Dendrobium* and *Bulbophyllum* (Freudenstein and Senyo 2008). However, Barrett and Davis identified functional *matK* copies in the plastomes of *Corallorhiza* (Barrett and Davis 2012). In this study, all Monotropoideae and Burmanniaceae species completely retained the *matK* gene, whereas it was only partially retained in Orchidaceae, suggesting that the importance of *matK* for plastome function varies across different lineages. It is currently widely accepted that MatK participates in post‐transcriptional processing of group IIA intron‐containing genes. The absence of *matK* in parasitic *Cuscuta* species correlates with the loss of introns requiring MatK activity. Notably, although *M. odorata* contains *matK*, its plastome lacks group IIA intron‐containing genes. Conversely, despite the absence of matK in *Rafflesia gardneri*, its plastome retains three group IIA introns, two of which have been confirmed to be correctly spliced and can be bound by MatK in tobacco (Zoschke et al. 2010). These results may indicate that the relationship between matK and group IIA intron‐containing genes is not strictly obligatory. Given the differential loss of *matK* in Orchidaceae alongside its convergent retention in distantly related species (ranging from monocots to eudicots), we propose that such patterns of gene loss and retention primarily reflect convergent evolution among species rather than shared evolutionary history. Therefore, we suggest that over sufficiently long time scales, non‐photosynthetic organisms tend to converge toward similar, or even identical, plastid genome architectures, and the characteristics of these genomes provide clues about the evolutionary constraints governing plastid gene loss.

In contrast to the widespread loss of photosynthesis and transcription/translation‐related genes in fully mycoheterotrophic plants, the *accD* gene, encoding the beta subunit of the carboxyltransferase component of acetyl‐CoA carboxylase, is frequently retained due to its fundamental function. This enzyme catalyzes the first committed step in fatty acid biosynthesis, producing precursors essential for constructing all cellular membrane systems, including the plastid's own inner membranes. Although mycoheterotrophic plants have completely lost photosynthetic capacity, their plastids persist as organelles and require basic membrane structures to maintain any residual functions (e.g., limited fatty acid synthesis). Consequently, natural selection favors the in situ retention of *accD* in the plastid genome as the most cost‐effective way to maintain the basic capacity for endomembrane construction (Delannoy et al. 2011; Wicke et al. 2011). The widespread retention of core housekeeping genes such as *accD*, *matK*, and *clpP* contrasts sharply with the systematic loss of atp and photosynthesis‐related genes. This precisely illustrates the “economy principle” governing plastid functional degradation. Only when the function of a gene or gene class becomes completely redundant due to a radical lifestyle shift, such as photosynthesis, does it succumb to loss under relaxed selection. Conversely, genes still critical for basic organelle survival, like *accD*, remain under purifying selection and are retained even in highly degraded genomes. However, exceptions exist. Recent studies have revealed that *B. fungosa* has completely abandoned its plastid genome, with genes including *accD* transferred to the nuclear genome (Molina et al. 2014; Chen et al. 2023). The case of *Balanophora*, in which the plastid genome has been completely lost and its functions (e.g., *accD, clpP*) transferred to the nuclear genome (Molina et al. 2014; Chen et al. 2023), represents an extreme outcome within the continuum of plastid genome reduction. However, whether this represents a true ‘endpoint’ of parasitic plant evolution or simply one of multiple possible outcomes under strong relaxed selection remains an open question. The diversity of plastome configurations observed across mycoheterotrophic and parasitic lineages suggests that multiple ‘end states’ may exist, depending on lineage‐specific functional constraints and the timing and mode of gene transfer to the nucleus.

In summary, the degradation of plastid genomes in mycoheterotrophic plants is a complex evolutionary process driven by strong relaxed selection, following a functional priority order yet exhibiting lineage‐specific idiosyncrasies. The main trajectory adheres to the economic principle of “functional redundancy leading to gene loss”: from the early loss of photosynthesis‐related genes (*psa*/*psb*, *rbcL*), through the dissolution of the transcription‐translation apparatus (*rpo*), to the abandonment of energy production modules (*atp*), and potentially culminating in the loss of core housekeeping genes (*clpP*, *matK*). This main path validates the predictive power of the Barrett and Davis model and reveals the potent force of convergent evolution. However, the endpoints of degradation are diverse and constrained by residual lineage‐specific requirements. Due to differing biological contexts, the ultimate configuration of plastid degradation across lineages presents as a multi‐branched “spectrum” rather than a single endpoint. A lineage's position on this spectrum depends on its specific, remaining organellar functional needs. For example, orchids may retain *ycf1*/*ycf2* due to unknown requirements, or perhaps they are still en route to a fully mycoheterotrophic lifestyle. In contrast, *B. fungosa* represents the ultimate “outsourcing” solution, having transferred even *accD* to the nuclear genome, thereby completely discarding its plastid genome.

## 5 Conclusion

We sequenced and assembled the complete plastome of *M. callistoma*, providing the first plastome record for this species. The *29,784‐bp* plastome has a GC content of 30.8%, contains 40 genes (20 PCGs, 16 tRNAs, and four rRNAs), and lacks the canonical quadripartite structure. Comparative analysis of 30 mycoheterotrophic plastomes revealed extreme contraction in *Sciaphila* and *Mitrastemon*, whose plastomes are approximately 20 kb long, and a weak positive association between GC content and genome size. Within Monotropoideae, plastomes were smaller and less syntenic in more recently *diverged lineages*, and all sampled *Monotropa* species lacked *rps23*, suggesting a lineage‐specific loss. Phylogenomic analysis strongly supported Monotropoideae and resolved the sampled relationships among its 10 genera, with *Pterospora* and *Sarcodes* forming successive early branches. Divergence‐time and ancestral‐range analyses support an Asian origin for Clade 1 during the late Cretaceous to early Cenozoic, followed by dispersal to North America through *Beringia*. The *Monotropsis* + *Monotropastrum* + *Monotropa* lineage subsequently appears to have dispersed back to Asia during the late Eocene to early Oligocene, giving rise to East Asian endemics including *M. humile* and *M. callistoma*. These findings clarify plastome reduction and phylogenetic relationships in Monotropoideae while supporting a complex history of intercontinental dispersal. Because *Cheilotheca* and *Eremotropa* were not sampled, their plastomes will be important for completing the evolutionary framework of the subfamily.

## Author Contributions

**Guocui Deng:** formal analysis (equal), writing – review and editing (equal). **Kaili Duan:** formal analysis (supporting), writing – review and editing (supporting). **Niyan Xiang:** data curation (lead), resources (lead). **Feng Gao:** software (equal). **Peng Zhan:** data curation (equal), resources (lead). **Rui Shu:** investigation (lead). **Qingsong Lin:** data curation (equal). **Qing Zuo:** writing – review and editing (equal). **Tao Yuan:** formal analysis (lead), writing – original draft (lead), writing – review and editing (lead).

## Funding

This study was supported by Xinjiang Uygur Autonomous Region Natural Science Foundation Youth Science Fund Project (2026D01C314/2026D01C27).

## Conflicts of Interest

The authors declare no conflicts of interest.

## Supporting information


**Figure S1:** Synteny analysis of Monotropoideae plastomes. Collinear blocks are connected by colored bars, with *Pterospora andromedea* as the reference species in the upper‐left corner of each panel. Color intensity indicates sequence similarity, with red denoting the highest similarity.

**Table S1:** Species and accession numbers used in the comparative genomic analysis of Ericaceae family.
**Table S2:** Fossil information.
**Table S3:** Species and distribution site codes included in the ancestral distribution analysis.

## Data Availability

The datasets generated and analyzed during the current study are available in the Figshare repository (https://doi.org/10.6084/m9.figshare.31436140). The data supporting the findings of this study are openly available on GitHub at https://github.com/taotaoyuan/phylogenetic‐relationships‐within‐Monotropoideae.
